# On the Relationship Between Sensory Eye Dominance and Stereopsis in the Normal-Sighted Adult Population: Normative Data

**DOI:** 10.3389/fnhum.2018.00357

**Published:** 2018-09-07

**Authors:** Yonghua Wang, Lele Cui, Zhifen He, Wenman Lin, Jia Qu, Fan Lu, Jiawei Zhou, Robert F. Hess

**Affiliations:** ^1^Department of Ophthalmology, The First Affiliated Hospital of Wenzhou Medical University, Wenzhou, China; ^2^School of Ophthalmology and Optometry and Eye Hospital, and State Key Laboratory of Ophthalmology, Optometry and Vision Science, Wenzhou Medical University, Wenzhou, China; ^3^Department of Ophthalmology, McGill Vision Research, McGill University, Montreal, QC, Canada

**Keywords:** sensory eye dominance, stereopsis, binocular function, normal-sighted population, contrast

## Abstract

The extent of sensory eye dominance, a reflection of the interocular suppression in binocular visual processing, can be quantitatively measured using the binocular phase combination task. In this study, we aimed to provide a normative dataset for sensory eye dominance using this task. Based on that, we also assessed the relationship between perceptual eye dominance and stereopsis. One-hundred and forty-two adults (average age: 24.00 ± 1.74 years old) with normal or corrected to normal monocular visual acuity (logMAR < 0.00) participated. Observer’s sensory eye dominance was quantified in two complementary ways: the interocular contrast ratio when the two eyes were balanced (i.e., the balance point) and the absolute value of the binocular perceived phase when each eye viewed maximum contrast stimuli in binocular phase combination task. Stereo acuities were measured with maximum contrast stimuli using an identical spatial frequency (0.30 cycles/degree) and stimulus arrangement to that used in the eye dominance assessment. The averaged balance point was 0.93 ± 0.06 (Mean ± SD), the averaged absolute value of the binocular perceived phase when both eyes viewed maximum contrast stimuli was 7.62 ± 5.91°, and the averaged stereo acuity was 2.19 ± 0.34 log arc seconds. Neither of these two sensory eye dominance measures were significantly correlated with stereo acuity (Balance point: *ρ* = 0.14, *P* = 0.10; Phase: *ρ* = −0.13, *P* = 0.13). The sensory eye dominance, as reflected using a phase combination task, and stereopsis are not significantly correlated in the normal-sighted population at low spatial frequencies.

## Introduction

Information from our two eyes does not necessarily have equal weighting at the level where they are combined in the visual cortex. The extent to which one eye’s input dominates is referred to as “sensory eye dominance.” Traditionally within the clinical literature, ocular dominance has been assessed using the sighting dominance test (Porac and Coren, 1976; Ehrenstein et al., 2005; e.g., the hole-in-the-card test (Dane and Dane, 2004)) and the Worth-4-dot test (Mustonen et al., 1993) in clinical practice. These tests give only a qualitative measure. In recent years, several laboratory-based techniques have been developed to quantitatively measure the sensory eye dominance of different individuals, these include the binocular rivalry task using gratings (Ooi and He, 2001; Handa et al., 2004, 2006, 2012), letters (Kwon et al., 2015) or noise patterns (Yang et al., 2010), the binocular phase combination task (Ding and Sperling, 2006; Huang et al., 2009), the dichoptically presented global motion coherence task (Hess et al., 2007), the dichoptically presented global orientation coherence task (Zhou et al., 2013a) and the binocular orientation combination task (Yehezkel et al., 2016). These studies, using different stimuli and paradigms, provide a more quantitative measure based on a computational framework of the known excitatory and inhibitory interocular networks (Ding and Sperling, 2006; Meese et al., 2006).

The binocular phase combination that was developed by Ding and Sperling (2006) is assumed to mainly target the interocular suppressive process in the primary visual cortex (Huang et al., 2010), where the left and right eye stimulus phases are processed by binocular neurons (Anzai et al., 1999). This task is of particular interest for researchers and clinicians who study normal and abnormal binocular processing at the level of the primary visual cortex. Several models have been developed to explain the binocular phase combination processing, for normal adults (Ding and Sperling, 2006; Ding et al., 2013; Zhou et al., 2014), for patients, e.g., amblyopia (Huang et al., 2009, 2011; Ding et al., 2013) and for interocularly imbalanced luminance viewing conditions, e.g., monocular luminance deprivation (Zhou et al., 2013b). Using this task, it has been demonstrated that abnormal sensory eye dominance occurs in patients with amblyopia (Huang et al., 2009; Ding et al., 2013), strabismus (Kwon et al., 2014), anisometropia (Zhou et al., 2016), surgically-corrected intermittent exotropes (Feng et al., 2015), surgically-corrected strabismic patients (Zhou et al., 2017), treated amblyopes (Chen et al., 2017; Zhao et al., 2017) and LASIK-corrected anisometropes (Feng et al., 2017).

In all the above-mentioned clinically related studies, the normal sensory eye dominance is either assumed to be 1.00 (perfect balance) or assessed using small samples (2–40) of normal controls. However, there is so far no database of normal-sighted healthy adults’ sensory eye dominance using this quantitative laboratory technique. To facilitate the use of binocular phase combination paradigm in measuring individuals’ sensory eye dominance in the clinic, we set out, in this study, to provide a normative database (*n* = 142) for sensory eye dominance by deriving two complementary measures from the standard binocular phase combination paradigm (Ding and Sperling, 2006; Huang et al., 2009): the interocular contrast ratio when the two eyes were balanced (i.e., the balance point) and the absolute value of the binocular perceived phase when each eye viewed maximum contrast stimuli in binocular phase combination task.

Like sensory eye dominance, stereopsis, or depth perception based on the binocular disparity derived from the images projected onto the two retinas from the surrounding world, is also an important binocular function, one that plays a vital role in accurate hand-eye work and 3D perception (Melmoth et al., 2009). Recently, we showed that the stereo acuities of both the general population and the student population (around 20 years of age) are broadly distributed extending over a range of 2.00 or more log units, and as many as 30% have moderate to poor stereo (Hess et al., 2015). Similar to the clinical tests, such as TNO (Bosten et al., 2015), RDSs (Zaroff et al., 2003) and Frisby (Bohr and Read, 2013), the visual stimuli used were broadband. Because our sensitivity for stereopsis is spatial frequency dependent (Reynaud et al., 2013), we thus wanted to reassess this finding at a fixed spatial frequency. To simplify the test, and to make it easier for any further comparison of the two binocular processing measures (i.e., binocular phase combination and stereopsis), we measured stereopsis in 142 normal-sighted adults using stimuli of identical low spatial frequency, contrast and stimulus arrangement as that used for the binocular phase combination task. We ask two separate questions, first, how are sensory eye dominance and stereo acuity distributed within the normal-sighted population? and second, do subjects with well-balanced eyes have better stereopsis? Both were evaluated at low spatial frequency (0.30 cycles/degree).

## Materials and Methods

### Participants

One-hundred and forty-two adults (average age: 24.00 ± 1.74 years old) with normal or corrected to normal monocular visual acuity (logMAR < 0.00) were recruited in this study. Subjects wore their habitual optical correction (refractive errors ranging from Plano to −7.50 D) if necessary. All cases had no previous history of binocular dysfunction or ocular surgery and had anisometropia of less than 1.50 D. Except for one of the authors (YW), all observers were naive as to the purpose of the experiment. This study was carried out in accordance with the recommendations of the ethics committee of the Wenzhou Medical University, with written informed consent from all subjects after explanation of the nature and possible consequences of the study.

### Apparatus

All measurements were conducted on a PC computer running Matlab (MathWorks Inc., Natick, MA, USA) with Psychtoolbox 3.0.9 extensions (Brainard, 1997; Pelli, 1997). The stimuli were dichoptically presented by head-mount Z800 pro goggles (eMagin Corp., Washington, CA, USA), which was driven by a Dual-Head2Go display adaptor (Matrox Electronic Systems Ltd., Dorval, QC, Canada) and had a simulated viewing distance of 3.60 m, a refresh rate of 60 Hz, a resolution of 800 × 600 (pixels per degree = 26.40) and a mean luminance of 160 cd/m^2^ in each eye. The Z800 pro goggles contain 2 OLED screens for the two eyes, which had a linear luminance response thus no gamma-correction was needed.

### Design

**Figure 1:**
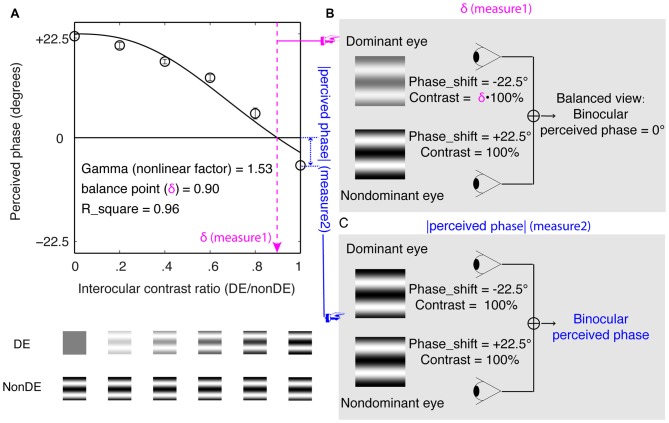
Experimental design and visual stimuli. **(A)** The results of one participant. The horizontal axis represents the interocular contrast ratio (dominant eye/nondominant eye). The vertical axis represents the binocular perceived phase. The solid line represents a curve fit using the contrast-gain control model (Ding and Sperling, 2006; Huang et al., 2009). In which, “*balance point*” and “*gamma*” are two free parameters. “*balance point*” represents the interocular contrast ratio when the two eyes make equal contributions to binocular combination and “*gamma*” represents a nonlinear factor. **(B)** An illustration of balance point measure of the sensory eye dominance. Observers’ sensory eye dominance was quantified by the interocular contrast ratio when the two eyes were balanced (i.e., when the binocular perceived phase was 0°). **(C)** An illustration of binocular perceived phase measure of the sensory eye dominance. Observers’ sensory eye dominance was quantified by the absolute value of the binocular perceived phase when each eye viewed maximum contrast stimuli.

The sensory eye dominance was quantitatively assessed by a binocular phase combination paradigm (Ding and Sperling, 2006; Huang et al., 2009). In this measure, two horizontal sine-wave gratings (0.30 cycles/degree), with opposite vertical phase-shift of +22.50° and −22.50° related to the horizontal meridian, were dichoptically presented to the two eyes. Observers’ sensory eye dominance was quantified in two complementary ways: the averaged interocular contrast ratio when the two eyes were balanced (i.e., the balance point; Figure 1B) and the binocular perceived phase when each eye viewed maximum contrast stimuli (Figure 1C). These two measures are illustrated in Figure 1.

In particular, we measured the binocular perceived phase when the nondominant eye had a fixed contrast of 100% and the dominant eye had a contrast of 100% × (0, 0.20, 0.40, 0.60, 0.80 and 1.00). We fitted the binocular perceived phase vs. the interocular contrast ratio curve, using the contrast-gain control model, in which each eye exerts gain control on the other eye’s gain control in proportion to the strength of its own input (Ding and Sperling, 2006; Huang et al., 2009). Figure 1A shows an individual subject’s data with curve fits using the contrast-gain control model (Ding and Sperling, 2006; Huang et al., 2009).

For each interocular contrast ratio, two configurations were used in the measurement to cancel any potential positional starting bias: in one configuration, the phase-shift was +22.50° in the dominant eye and −22.50° in the nondominant eye, in the other, the reverse. Different conditions (configurations and interocular contrast ratios) were randomized in different trials in one test. The perceived phase of the cyclopean grating at each interocular contrast ratio was quantified by half of the difference between the measured perceived phases in these two configurations. The perceived phase and its standard error were calculated based on eight repetitions in one test.

**Figure 2:**
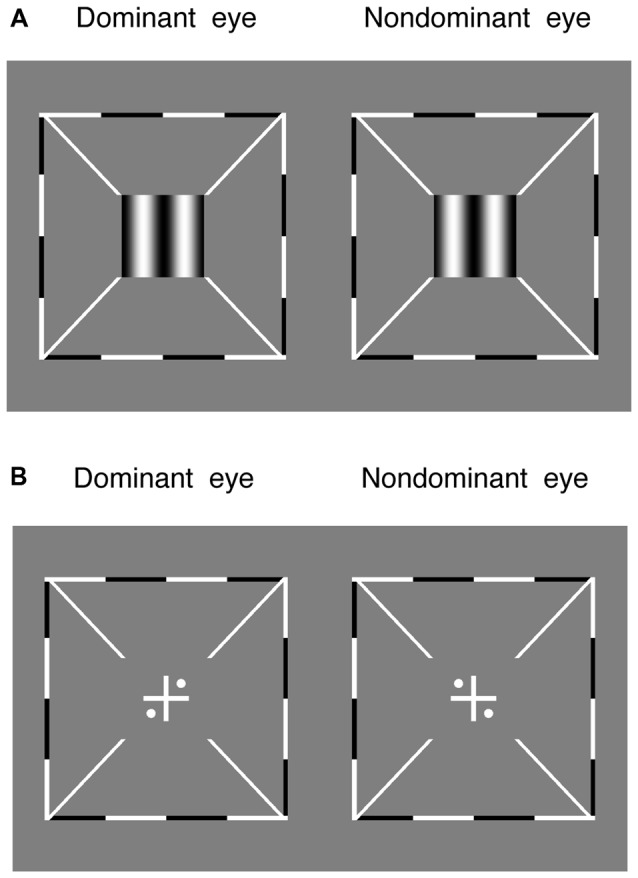
The alignment task and the stereo test. **(A)** Two maximal contrast gratings, with equal and opposite phase-shift of “offset/2” relative to the vertical meridian, were dichoptically presented to the two eyes. Observers were asked to answer whether the perceived grating was in front or behind the screen. **(B)** Observers were asked to adjust the relative positions of the images to make sure the two eyes were aligned. Surrounding all gratings, a high-contrast frame (thickness, 0.11°; length, 6°) with four white diagonal lines (thickness, 0.11°; length, 1.11°) was always presented during the two tests to help observers maintain fusion.

Stereo acuity was measured with a 3-down-1-up staircase method to determine the threshold offset in the depth discrimination task. In this task, two maximal contrast (100%) vertical gratings (0.30 cycles/degree), with opposite horizontal phase-shift of “±offset/2” relative to the vertical meridian, were dichoptically presented to the two eyes for 1 s (Figure 2A). The direction of the phase-shift was randomly set in different trials. Observers were asked to answer whether the perceived grating was perceived to be in front or behind the screen. The offset was initially set based on individuals’ performance in the pilot test and controlled by a 3-down-1-up staircase method in the following trials. The staircase had a relative step size of 50% in the first trial and 25% thereafter. Each staircase was repeated three times and the last six reversals of each repetition were averaged to obtain the threshold for that staircase (i.e., 18 reversal points in total).

Both the sensory eye dominance and the threshold offset were measured twice in 2 days and the results were averaged based on these two repetitions. Before the start of data collection, eye alignment (Figure 2B) was provided to ensure the fusion of two eyes; proper demonstrations of the tasks were provided by practice trials to ensure observers understood the tasks. During the test, observers were allowed to take short-term breaks whenever they felt tired.

### Procedure

In measuring the sensory eye dominance, observers were asked to adjust the reference line to coincide with the perceived center of the black stripe located in the middle of the binocularly combined grating. The gratings were presented continually until subjects made their decision by pressing the space bar and then followed by a 500-ms blank (with only the surrounding frame and diagonal bars presented) and the presentation of the next trial. In the stereo test, observers were asked to answer whether the perceived grating was in front of or behind the screen. The gratings were presented for 1 s, which followed by the eye alignment stimuli (Figure 2B) that were presented continuously until subjects made their decision by pressing a corresponding key and then followed by a 500-ms blank (with only the surrounding frame and diagonal bars presented) and the presentation of the next trial.

## Results

**Figure 3:**
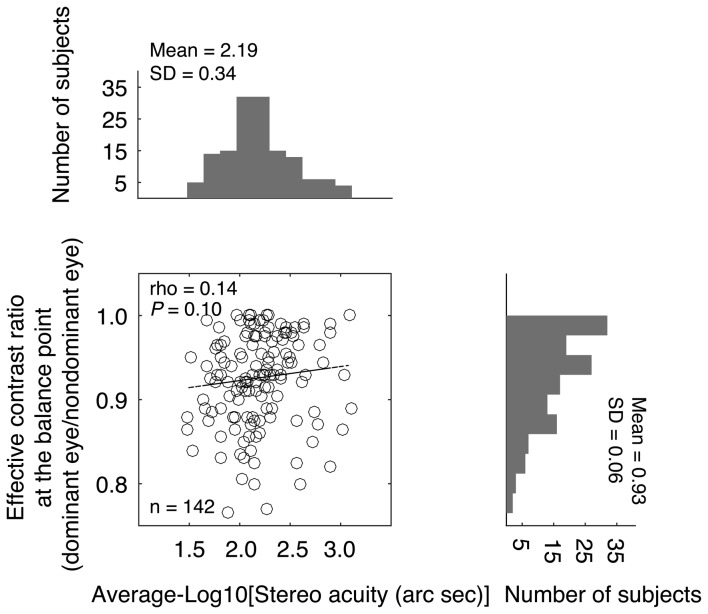
Relationship between the effective contrast ratio at the balance point and the stereo acuity. In the scatter plot, each point represents one observer; Spearman’s rank correlation coefficient is provided in the figure. The histogram of the effective contrast ratio at the balance point and the histogram of the stereo acuity are also provided.

The effective contrast ratio at the balance point (dominant eye/non dominant eye) is plotted as a function of the stereo acuity for the 142 normal-sighted participants in Figure 3, with histograms for the two measures plotted on the sides. It should be noted that for 11 observers (7.70%), their fitted effective contrast ratio at the balance point (for short, “balance point”) was larger than 1, which indicated reversed eye dominance in binocular phase combination, i.e., their pre-assigned “dominant eye” from the sighting dominance test (Porac and Coren, 1976; Ehrenstein et al., 2005) was actually less dominant in the binocular phase combination measure. To simplify the analysis, we used the reciprocal value as their balance point. The averaged balance point was 0.93 ± 0.06 (Mean ± SD; the 95% confidence interval was from 0.92 to 0.94; median was 0.93; IQR was 0.09). A Shapiro-Wilk test showed that the balance points were not normally distributed (*P* < 0.001). The averaged stereo acuity was 2.19 ± 0.34 log arc seconds (the 95% confidence interval was from 2.13 to 2.24; median was 2.15; IQR was: 0.39). Stereo acuities were also not normally distributed (*P* = 0.03). A Spearman’s *ρ* correlation test showed that the effective contrast ratio at the balance point and the stereo acuity value were not significantly correlated (*ρ* = 0.14, *P* = 0.10).

**Figure 4:**
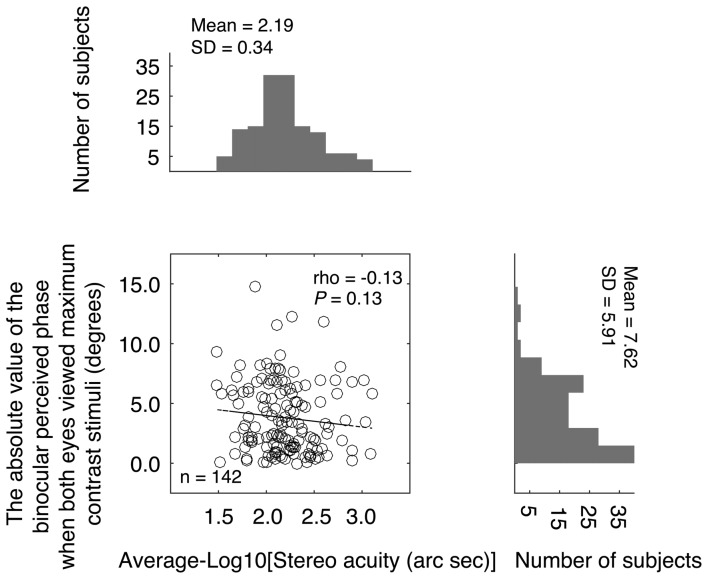
Relationship between the absolute value of the binocular perceived phase when both eyes viewed maximum contrast stimuli and the stereo acuity. In the scatter plot, each point represents result of one observer; Spearman’s rank correlation coefficient is provided in the figure. The histogram of the absolute value of the binocular perceived phase when both eyes viewed maximum contrast stimuli and the histogram of the stereo acuity are also provided.

On criticism of the above comparison is that while stereo acuity was measured for stimuli of the same high contrast, the balance point was measure for stimuli of potentially different contrast because the nondominant eye viewed a stimulus of maximum contrast stimuli while the dominant eye viewed a reduced contrast stimulus (see Figure 1A). The different interocular stimulus contrast arrangements in the two measures might have affected their comparison. To better show the relationship between the binocular phase combination and the stereopsis, we also quantified the sensory eye dominance using another method, namely, the absolute value of the binocularly perceived phase measurement when both eyes viewed maximum contrast stimuli. In this measure, the absolute value of the binocularly perceived phase ranges from 0° (means the two eyes were balanced in binocular phase combination) to 22.50° (means one eye totally dominant). The advantage of this comparison is that the interocular contrasts are identical for the two measures we want to compare. In Figure 4, we plotted the absolute value of the binocularly perceived phase when both eyes viewed maximum contrast stimuli as a function of the stereo acuity for the 142 observers. The histograms for these two tests were also provided on the sides of the graph. The averaged absolute value of the binocular perceived phase when both eyes viewed maximum contrast stimuli was 7.62 ± 5.91°(the 95% confidence interval was from 6.64 to 8.59; median was 6.25; IQR was 9.50), which was also not normally distributed (*P* < 0.001). A Spearman’s *ρ* correlation test revealed that the absolute value of the binocular perceived phase when both eyes viewed maximum contrast stimuli and the stereo acuity value were also not significantly correlated (*ρ* = −0.13, *P* = 0.13).

## Discussion

In this study, we quantitatively assessed sensory eye dominance using the binocular phase combination task in a sample of 142 normal-sighted healthy adults using two complementary measures as illustrated in Figures 1B,C. We show that the averaged sensory eye dominance was 0.93, which was close to unity (i.e., perfect binocular balance); neither the balance point (*ρ* = 0.14, *P* = 0.10) nor the absolute value of the perceived phase when the two eyes viewed maximum contrast stimuli (*ρ* = −0.13, *P* = 0.13) was significantly correlated with stereo acuity. Within the normal-sighted population, having more balanced eyes does not ensure better stereopsis. Our results further support a recent report from Wu et al. (2018), who found no correlation between sensory eye dominance as measured with the continuous flashing technique and stereopsis as measured with random dots on the same CRT monitor used for ocular dominance test.

Only 18 of 142 (i.e., 12.70%) observers had a balance point of exactly 1.0 (i.e., balanced eyes), while the others (i.e., 87.30%) had different extents of binocular imbalance. Some cases exhibited a balance point of 0.90 or less, indicating that the dominant eye was balanced with the non dominant eye when the interocular contrast ratio was 0.90 or smaller. These results are consistent with several studies that have measured sensory eye dominance using other quantitative techniques: Jiang et al. (2015) found that 61.30% of non-anisometropic subjects showed strong ocular dominance using a continuous flashing technique; Li et al. (2010) found that binocular imbalance in 44 normal individuals was bimodally distributed using a dichoptic motion coherence threshold test, with 61% of them having a weaker dominance and the remainder having a stronger imbalance; Handa et al. (2006) found 71.70% of 60 normal participants and 80% of 10 cataract patients had weaker binocular imbalance using the binocular rivalry test; Yang et al. (2010) showed that 62% of observers among 88 adults had a weak eye dominance using a dichoptic continuous flash suppression paradigm.

We assessed the relationship between sensory eye dominance and stereopsis in normal adults. We designed these two tasks around a common stimulus so that comparisons between sensory eye dominance and stereopsis would be valid. In particular, we used the same spatial frequency and a comparable stimulus arrangement for studying these two visual functions. Furthermore, we show that neither the effective contrast ratio at the balance point (dominant eye/non dominant eye) nor the absolute value of the binocular perceived phase, when both eyes viewed maximum contrast stimuli, was significantly correlated with the stereopsis. This makes sense since the current study relies on normative dataset in normal adults, whose within-subject variability is relatively small compared with patients with disorders of binocular vision (e.g., amblyopia, strabismus) or other age groups. Previous studies have shown that, for patients with amblyopia, the stronger the departure from normal sensory eye dominance, as assessed by dichoptically presented global motion coherence, the poorer the stereo acuity measured with the standard clinical book tests (Li et al., 2011). Furthermore, dichoptic visual training designed to strengthen fusion, and as a consequence, reduce suppression, has been shown to significantly improve stereopsis (Li et al., 2013). On the other hand, Feng et al. (2015) found that treated intermittent exotropes still had a significant degree of abnormal sensory eye dominance (measured with the binocular phase combination task) even though they had near to normal stereo acuity (measured with clinical book tests), which indicates that in some cases, the sensory eye dominance might not tightly correlate with the stereopsis. The inconsistent relationship between sensory eye dominance and stereopsis in these different studies might be due to small numbers of subjects used (*n* = 3–43), different stimulus spatial frequency ranges used in the comparison of these two measures and/or different disparity resolutions or non-stereoscopic cues in different tests, e.g., TNO, Titmus, Lang, Frisby, or other in-house developed tests (Simons, 1981; Hall, 1982; Garnham and Sloper, 2006). Nevertheless, our study provides potentially valuable normative data for these cases in studying the limitations of sensory eye dominance and stereo acuity.

Last but not least, when referring to our value of stereo acuity an important proviso must be made, namely that our measurements were made at a low spatial frequency (0.30 cycles/degree), as this gave us the required spatial resolution to make accurate phase measurements for the sensory eye dominance task. Our conclusions are thus limited to this spatial scale. Our stereo thresholds are around 150 arc seconds which is not a particularly high stereo acuity (normally expected to be around 10–20 arc seconds) but this is simply due to the spatial frequency used; the higher the spatial frequency the better the stereo acuity (Schor and Wood, 1983). For our 0.30 cycles/degree stimulus, the stereo acuity is in line with what is expected (Hess et al., 2002). Similar to our previous observation (Hess et al., 2015), the stereo acuities are broadly distributed extending over a range of 2.00 log units. It is surprising that stereopsis shows such individual variability compared with sensory eye dominance in normal-sighted young adults. Higher spatial frequencies should be tested in future studies for both the binocular balance and the stereopsis. Nevertheless, our study shows clear evidence that, at low spatial frequency, normal-sighted adults with balanced eyes in binocular viewing may still have moderate to poor stereo. For these cases, binocular training on stereo processing, rather than a binocular approach targeting the interocular suppression (Gao et al., 2018), should be encouraged to improve their stereo acuity.

## Author Contributions

JZ, LC, FL, JQ and RH conceived the experiments. YW and ZH performed the experiments. YW, WL, LC and JZ analyzed the data and interpreted the data. YW, JZ and RH wrote the manuscript. All authors reviewed the manuscript.

## Conflict of Interest Statement

The authors declare that the research was conducted in the absence of any commercial or financial relationships that could be construed as a potential conflict of interest.
